# The Dissociation of Gefitinib Trough Concentration and Clinical Outcome in NSCLC Patients with EGFR Sensitive Mutations

**DOI:** 10.1038/srep12675

**Published:** 2015-07-31

**Authors:** Shuang Xin, Yuanyuan Zhao, Xueding Wang, Yan Huang, Jing Zhang, Ying Guo, Jiali Li, Hongliang Li, Yuxiang Ma, Lingyan Chen, Zhihuang Hu, Min Huang, Li Zhang

**Affiliations:** 1 Institute of Clinical Pharmacology, School of Pharmaceutical Sciences, Sun Yat-Sen University, Guangzhou, PR China; 2 Department of Medical Oncology, Sun Yat-Sen University Cancer Center, State Key Laboratory of Oncology in South China; Collaborative Innovation Center for Cancer Medicine, Guangzhou, PR China; 3 Department of Oncology, the First Affiliated Hospital of Guangzhou University of Traditional Chinese Medicine; 4 Clinical Trial Center, Sun Yat-Sen University Cancer Center, State Key Laboratory of Oncology in South China; Collaborative Innovation Center for Cancer Medicine, Guangzhou, PR China

## Abstract

Gefitinib is an essential drug for NSCLC patients harboring EGFR sensitive mutations. The approved dose 250mg/day is based on limited clinical trials, this research aims to explore the relationship between drug exposure and gefitinib response. C_trough_ of 87 NSCLC patients harboring EGFR sensitive mutations were determined by LC-MS/MS. The median of C_trough_ was 173.9 ng/ml (*P*_25_–*P*_75_, 130.5–231.2 ng/ml), and cutoff value 200 ng/ml was determined by X-Tile. The PFS between C_trough _< 200 ng/ml and C_trough _≥ 200 ng/ml groups were not significantly different (17.3 VS 14.8 months; *p *= 0.258). C_trough_ was not significantly associated with rash, diarrhea and hepatotoxicity. Non-smokers enjoyed longer PFS than smokers (18.7 VS 9.3 months; *p *= 0.025). The results showed that, for NSCLC patients with EGFR sensitive mutations, the PFS in lower trough concentration group were not inferior to that in higher trough concentration group and dose reduction is a rational suggestion for adjustment of dose regimen for aforementioned patients. More clinical trials are warranted to explore the precision dose schedule of gefitinib.

Gefitinib is an oral, reversible, tyrosine kinase inhibitor (TKI) of epidermal growth factor receptor (EGFR) that plays a key role in the biology of non small cell lung cancer (NSCLC). For patients with advanced NSCLC harboring EGFR-TKI sensitive mutations, using EGFR-TKI as first-line treatment, progression-free survival (PFS) was reported to be two to three times better than platinum-doublet chemotherapy^1^,^2^,^3^. Based on these results, EGFR-TKIs monotherapy has become a standard regimen for advanced NSCLC with EGFR mutations.

However, several questions need to be addressed regarding the approved dosage regimen (250mg/day) established based on limited clinical trials. According to the results of Iressa Dose Evaluation in Advanced Lung Cancer trial ı and trial ıı (IDEAL-1 and IDEAL-2), the doses 250 mg/day and 500 mg/day have comparable response rate in all the NSCLC patients^4^,^5^. Meanwhile, recently, a post hoc analysis from NEJ002 was conducted to examine the efficacy of dose-reduction gefitinib compared with that of standard-dose gefitinib in EGFR-mutated NSCLC patients. In this research, a dose reduction of gefitinib by changing the everyday schedule to every 2 days schedule was permitted when grade ıı toxicity was observed. Interestingly, the low-dose group showed not-inferior efficacy (response and survival) compared with standard-dose group^6^. However, Ichihara E. *et al.* reported that in EGFR-mutated NSCLC patients, PFS of the subjects with higher BSA was significantly worse than that of those with lower BSA and consequently postulated that blood concentrations were reversely correlated with BSA and then influenced the gefitinib response^7^. The postulations from Ichihara E.’s research are in contradiction with aforementioned clinical trials. To date, no study has been systemically conducted yet about the relationship between the gefitinib PKs and its effects in NSCLC patients with EGFR mutation. The dose regimen of 250 mg/day has not been optimized for this specific group of NSCLC patients.

Recently, the concept of “precision medicine” has been raised up to further discover inter-individual difference of therapy outcomes and provide the best available care for each individual^8^. For gefitinib, as aforementioned, available data to verify the relationship between dosage regimen, blood exposure and drug effects are limited in NSCLC patients with EGFR sensitive mutations. The objective of this study was to retrospectively explore the relationship between gefitinib trough concentration, and clinical outcomes in both efficacy and safety in patients carrying EGFR sensitive mutations.

## Results

### Patients’ characteristics

In total, 87 patients were included in the final analysis. Patient characteristics were summarized in Table 1. 80 patients had major EGFR mutations (exon 19 deletions or exon 21 L858R), 7 patients carried other sensitizing-mutations, such as exon 21 L861Q, exon 20 frame insertion. The median BSA was 1.66 m^2^ (range, 1.26–2.06 m^2^). The median age was 56 years (range, 29–79). The number of male and female was roughly comparable. Most patients had good PS (performance status) and carried adenocarcinoma histology. 23 (26%) patients were post-operatively relapsed patients. 24 (28%) patients were former or current smokers. 9 patients (10%) suffered from CNS metastases after gefitinib administration. Table 2 compared the characteristics of the patients who were treated with gefitinib in the first-line setting with those in the later-line setting.

### Association between clinicopathologic features and gefitinib trough concentration

The median of gefitinib trough concentration is 173.9 ng/ml (*P*_25_–*P*_75_, 130.5–231.2 ng/ml). For clinicopathologic features such as gender, BSA, PS, smoker, CNS metastases and age, no factor was significantly correlated with gefitinib trough concentration (Table 3). The association between the BSA and trough concentration was analyzed by Spearman rank correlation test, *r*_s _= −0.112 (*P *= 0.320). Gefitinib trough concentration was not correlated with CNS metastases. (Spearman rank correlation test, *r*_s _= −0.142, *P *= 0.194).

### Relationship between gefitinib trough concentration and objective response, progression-free survival

**Figure 1:**
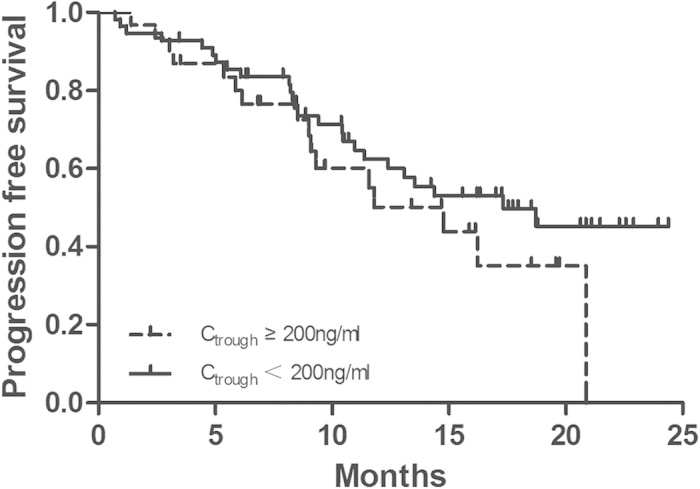
PFS of patients with C_trough _< 200 ng/ml (solid line) or C_trough _≥ 200 ng/ml (dashed line).

The objective response (complete response or partial response [PR]) rate is 57.5%. The disease control (complete response, partial response [PR] or stable disease [SD]) rate is 93.1%. In univariate analysis, gefitinib trough concentration was not a predictor of objective response. The log-rank test was used to compare progression-free survival in each subset of patients (Table 4). The PFS between C_trough _< 200 ng/ml and C_trough _≥ 200 ng/ml were not significantly different (17.3 VS 14.8 months; *p *= 0.258, Fig. 1).

### Relationship between clinicopathologic features and objective response, progression-free survival

**Figure 2:**
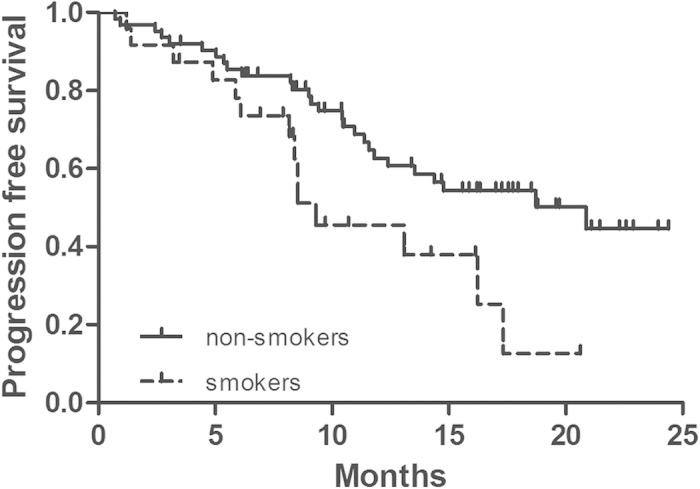
PFS of patients with non-smokers (solid line) or smokers (dashed line).

In univariate analysis, no factors were strong predictors of objective response. In log-rank test, non-smokers enjoyed longer progression-free survival than smokers (18.7 VS 9.3 months; *p *= 0.025, Fig. 2, Table 4).

### Correlation between gefitinib trough concentration and toxicity

In binary logistic regression, trough concentration did not significantly associate with rash, diarrhea and hepatotoxicity (Table 5).

## Discussion

Gefitinib is a standard therapy for NSCLC patients with EGFR sensitive mutations, however, few clinical trials have been conducted to discuss the association between gefitinib exposure and drug effect. Until now, whether the approved dose 250 mg/day is a rational dose schedule in patients with EGFR sensitive mutations remains disputable. We undertook this retrospective research to evaluate the impact of pharmacokinetic factors on gefitinib drug effect and adverse drug reaction in NSCLC patients with EGFR sensitive mutations. Our results indicated that, for patients with EGFR sensitive mutations, after using gefitinib 250 mg/day, the progression-free survival of C_trough _< 200 ng/ml group were not inferior to that in higher trough concentration (C_trough  _≥ 200 ng/ml) group. Meanwhile, trough concentration was not significantly associated with the adverse effects, including skin rash, diarrhea and hepatotoxicity. What’s more, non-smokers enjoyed better progression free survival. To our knowledge, this research provides the first evidence that the inter-patient pharmacokinetic variability is not the major cause of variation in gefitinib responses in patients with EGFR sensitive mutations.

One of the characteristics for molecularly targeted anticancer agents, the dose and response in efficacy and safety are not tightly correlated^9^,^10^. Regarding to gefitinib, 250 mg/day had a comparable response rate to 500 mg/day and milder adverse effects, in the Iressa Dose Evaluation in Advanced Lung Cancer (IDEAL-1 and –2) trials^4^,^5^. The results from NEJ002 study are similar, in which the progression-free survival and overall survival of dose-reduction group tended to be better, or at least similar to those of the standard-dose group^6^. Our results further confirmed this phenomenon. Recently, a retrospective study, focused on the relationship between BSA and PFS, demonstrated that for patients harboring EGFR mutations, the median PFS of the patients with higher BSA (≥1.5 m^2^) was significantly worse than that of those with lower BSA (<1.5 m^2^) (10.4 VS 18.0 months; *p *= 0.019, log-rank test). The authors postulated that blood concentrations were reversely correlated with BSA and then influenced the gefitinib response^7^. However, this postulation has not been confirmed in our study. In our research, the BSA and trough concentration were not significantly correlated.

Further, both preclinical and clinical research revealed that the concentrations of gefitinib in tumor and skin were much higher than plasma concentrations in xenograft mice and patients, indicating potential tissue concentration of gefitinib^9^,^10^,^11^. In this research, the trough concentration ranged from 34.0 ng/mL to 503.0 ng/mL. According to literature, the ratio of tumor concentration to plasma concentration ranged from 1.12~250 to 1 (median 40 to 1), which demonstrated tremendous interindividual variability^12^. Haura EB *et al*. reported that the lowest tumor concentration detected in operative tumor was 3.638 μM^12^, which was much higher than gefitinib IC50 for growth in cell lines with EGFR sensitive mutations (gefitinib IC50 for H3255 cell lines = 0.04 μM)^13^. Consistent with the results from IDEAL1 and IDEAL2 clinical trials, our research further proved that, in patients with EGFR sensitive mutations, once plasma levels adequate to block tyrosine kinase have been achieved, additional plasma concentration escalations are unlikely to improve response.

Our research showed that non-smokers enjoyed longer PFS for patients with EGFR sensitive mutations. This is consistent with the results from several randomized trials^1^,^14^,^15^. Whole genome sequencing analyses have shown that lung cancer due to tobacco smoking was associated with a significant higher number of mutations per Mb (mutations per Mb: median 10.5, range 4.9–17.6) compared to never-smokers with lung cancer (mutations per Mb: median 0.6, range 0.6–0.9)^16^. The differences in mutation frequencies and the distinctive sets of mutations may contribute to potential difference in the predictive and/or prognostic significance according to smoking history. Future investigations into the predictive difference by smoking history may lead to the optimization of current targeted therapy and detailed mechanism researches about the eruption of smoking-related lung cancer are of great value for cancer therapy.

As a targeted therapy agent, the tolerability profile of gefitinib is better than previous cytotoxic agents, and the most common adverse events include rash, diarrhea, nausea and hepatotoxicity. Although Yuanyuan Zhao *et al.* found that in EGFR wide-type patients, those with C_trough  _≥ 200 ng/ml suffered more rash than with C_trough_ < 200 ng/ml^17^. In our research, trough concentration was not significantly associated with skin rash, diarrhea and hepatotoxicity in patients with EGFR sensitive mutations. To date, some studies have shown that the mechanisms of these adverse effects are considerably complex and need to be answered^18^,^19^,^20^,^21^. In terms of cutaneous toxicities, no significant correlation were seen between dose levels and EGFR signaling pathway inhibition in skin biopsies^9^. What’s more, the eruption of cutaneous adverse drug reaction results from not only the inhibition of EGFR pathway, which acts as an initiation stage and leads to abnormal proliferation, migration, and differentiation of keratinocytes^22^, but also the disruption of the integrity of the skin with the recruitment of inflammatory cells^23^. In conclusion, the blood concentration, in itself, is not enough to account for the interindividual variability of adverse drug reaction, indicating that it is the tissue specific concentration that results in related toxicity.

This research has some limitations. This type of study retrospectively analyzed heterogeneous data with regard to patient cohort and follow-up pattern, meaning that the study results seem speculative, not definitive. Therefore, our results should be interpreted cautiously. In addition, to date, a number of somatic mutations in the EGFR gene have been identified that are associated with increased activity of EGFR tyrosine kinase inhibitors, and inherited polymorphisms in the EGFR gene have been associated with altered EGFR expression or function, future study should consider all these factors in the enrolled subjects.

In conclusion, our retrospective analysis suggests that, under standard-dose gefitinib, patients with lower trough concentration may be clinically equivalent to patients with higher trough concentration for NSCLC patients with EGFR sensitive mutations. In NSCLC patients with EGFR mutation under the dosage of 250 mg/day, gefitinib PKs and its clinical outcome were not closely correlated. Thus, dose reduction is a rational suggestion for adjustment of dose schedule. Prospective clinical trials to clarify and explore the precision dose schedule for patients with EGFR sensitive mutation are warranted. Meanwhile, non-smokers enjoyed better progression free survival for this specific group of NSCLC patients.

## Material and methods

### Subjects

The main patient entry criteria included: age ≥ 18 years; histologically and cytologically proved NSCLC; Eastern cooperative oncology group performance status (ECOG PS) ≤ 2; adequate hematological, renal and hepatic functions; direct sequencing and Real-time PCR proved EGFR sensitive mutations.

Main exclusion criteria were as follows: uncontrolled systemic disease, any evidence of clinically active interstitial lung diseases, and other chemotherapy at the time of inclusion. The protocol was approved by the Ethical Committee of Cancer Center of Sun Yat-Sen University (CCSU), and written informed consent was obtained from each patient. The methods were carried out in accordance with the approved guidelines. This trial was registered with ClinicalTrials.gov, number NCT01994057 (date of registration: 2013/11/23).

BSA was calculated as follows: BSA (m^2^) = (body weight [kg]) ^0.425^ × (height [cm]) ^0.725^ × 0.007184

### Drug administration and disease assessment

Patients were treated with gefitinib monotherapy, 250 mg/day, until progression. Before study entry and prior to each subsequent treatment cycle (the first duration cycle is one month, then the rest of intervals for tumor reassessment are two months), routine laboratory tests (hematology and biochemistry assessments) and full tumor assessment with computed tomography (CT) scans or magnetic resonance imaging (MRI) scans were performed by the investigator and radiologists according to the response evaluation criteria in solid tumors [RECIST]. Objective responses (complete response or partial response [PR]) were confirmed 4 or more weeks after responses were first observed, stable disease [SD] was confirmed 4 weeks after responses were first observed. CNS metastases were recorded as progression disease. Adverse events (AEs) were recorded, graded for toxicity using the NCI-CTC, and assessed by the investigator.

### Detection of trough concentration and EGFR mutation

Plasma samples (2 mL) were collected on days 28 prior to drug administration, first tumor and toxicity assessment, frozen at −80  °C until analysis. The steady state trough concentration was analyzed with validated high-performance liquid chromatographic method with tandem mass spectrometric (LC-MS/MS)^24^. Details of the assay and partial validation data were included in the Supplementary material. EGFR mutation analysis was conducted using a primary tumor, metastatic lesion, such as lymph mode metastasis, or pleural effusion. EGFR mutation status was assessed by direct sequencing and real-time PCR (RT-PCR).

### Statistical analysis

Pharmacokinetic parameters were characterized by the use of descriptive statistics. The plasma gefitinib trough concentration (minimum steady-state concentration) was categorized into two groups: ‘low’ (trough concentration < 200 ng/ml) and ‘high’ (trough concentration ≥ 200 ng/ml). This trough concentration cutoff value was selected based on published data showing that a mean trough concentration of 0.40 μM (178.76 ng/ml) was achieved following oral dosing with gefitinib 250 mg^9^. Meanwhile, the cutoff value was validated by X-tile 3.4.7 independently^25^. Patients were further stratified according to gender (male versus female), smoking history (never vs. ever), tumor histology (adenocarcinoma versus non-adenocarcinoma), development of diarrhea or skin rash (yes vs. no) and trough concentration (‘high’ vs. ‘low’). The association between the potential predictive factors and trough concentration was analyzed by Spearman rank correlation test. Potential predictive factors were tested in univariate models (with chi-square tests or the Fisher exact test) to predict response to gefitinib. Kaplan–Meier analysis was used to estimate patient survival outcome. The log-rank test was used to compare the survival of patients with different predictive factors. Each analysis was two-sided, with a 5% significance level and a 95% confidence interval, and was performed with SPSS 13.0 for Windows software.

## Additional Information

**How to cite this article**: Xin, S. *et al.* The Dissociation of Gefitinib Trough Concentration and Clinical Outcome in NSCLC Patients with EGFR Sensitive Mutations. *Sci. Rep.* **5**, 12675; doi: 10.1038/srep12675 (2015).

## Supplementary Material

Supplementary Information

## Figures and Tables

**Table 1:** Patients’ characteristics.

**Characters**	**No. of patients**
EGFR mutation status	37/43/7
(exon19 deletions/exon21 L858R/other sensitive mutations)	
Median BSA, m^2^ (range)	1.66 (1.26–2.06)
Median age, years (range)	56 (29–79)
Smoker (smoker/non-smoker)	24/61
Gender (male/female)	47/40
Staging (recurrence/ ııı B or ıV)	23/64
ECOG PS (0–1/2–3)	87/0
Histology (Ad /non-ad)	80/7
CNS metastases	9/77

**Table 2:** The characteristics for qualitative variables of the patients treated with gefitinib in the first-line setting and in the later-line setting.

**Characters**	**Total (%)**	**First-line setting (%)**	**Later-line setting (%)**	P*
All patients	87 (100)	53 (61)	34 (39)	
EGFR mutation status				
exon19 deletions	37 (42)	19 (36)	18 (53)	0.235
exon21 L858R	43 (49)	30 (57)	13 (38)	
other sensitizing-mutations	7 (9)	4 (7)	3 (9)	
Gender				
Male	47 (54)	27 (51)	20 (59)	0.514
Female	40 (46)	26 (49)	14 (41)	
BSA,m^2^				
<1.66	40 (49)	24 (49)	16 (50)	1.000
≥1.66	41 (51)	25 (51)	16 (50)	
Age, year				
<60	51 (59)	28 (53)	23 (68)	0.189
≥60	36 (41)	25 (47)	11 (32)	
Smoker				
Smoker	24 (28)	11 (21)	13 (39)	0.086
Non-smoker	61 (72)	41 (79)	20 (61)	
Staging				
Recurrence	23 (26)	12 (23)	11 (32)	0.331
B or ı	64 (72)	41 (77)	23 (68)	
Histology				
Adenocarcinoma	80 (92)	51 (96)	29 (85)	0.105
Non-adenocarcinoma	7 (8)	2 (4)	5 (15)	
CNS metastases				
CNS metastases	9 (10)	7 (13)	2 (6)	0.472
Non-CNS metastases	77 (90)	46 (87)	31 (94)	

^*^Chi-square test and Fisher’s exact test, when there were fewer than five expected counts in the contingency table.

**Table 3:** Correlation between clinicopathologic features and gefitinib trough concentration

**Characters**	***r***_**s**_	***P***^a^
EGFR mutation status	0.076	0.485
exon19 deletions		
exon21 L858R		
other sensitizing-mutations		
Gender	−0.102	0.347
Male		
Female		
BSA,m^2^	−0.112	0.320
<1.66		
≥1.66		
Age, year	0.077	0.478
<60		
≥60		
Smoker	−0.028	0.831
Smoker		
Non-smoker		
Staging	−0.065	0.551
Recurrence		
B or ıV		
Histology	−0.165	0.127
Adenocarcinoma		
Non-adenocarcinoma		
Line	0.174	0.106
First-line setting		
Later-line setting		
CNS metastases	−0.142	0.194
CNS metastases		
Non-CNS metastases		

^*^P < 0.05;

^a^Spearman rank correlation test.

**Table 4:** Relationship between clinicopathologic features, gefitinib trough concentration and progression-free survival.

**Characters**	**Univariate analysis** ***P***^a^
Trough concentration	0.258
C < 200 ng/ml	
C **≥ **200 ng/ml	
EGFR mutation status	0.743
exon19 deletions	
exon21 L858R	
other sensitizing-mutations	
Gender	0.717
Male	
Female	
BSA, m^2^	0.591
<1.66	
≥1.66	
Age, year	0.483
<60	
≥60	
Smoker	0.025^*^
Smoker	
Non-smoker	
Staging	0.055
Recurrence	
ııı B or ıV	
Histology	0.074
Adenocarcinoma	
Non-adenocarcinoma	
Line	0.470
First-line setting	
Later-line setting	

^*^P < 0.05;

^a^Log-rank test.

**Table 5:** The correlation between gefitinib trough concentration and toxicity.

C_trough_	**Grade 0 V 1+**			**Grade 0 to 1 V 2+**		
	**OR**	**95% CI**	**P**	**OR**	**95% CI**	**P**
Rash	0.921	0.300–2.826	0.885	0.851	0.296–2.442	0.764
Diarrhea	1.461	0.488–4.377	0.498	0.974	0.083–11.435	0.983
Hepatotoxicity	1.111	0.332–3.715	0.864	0.301	0.034–2.640	0.279
