# Supplementary material for: The Dissociation of Gefitinib Trough Concentration and Clinical Outcome in NSCLC Patients with EGFR Sensitive Mutations
Source: Sci Rep. 2015 Jul 31;5:12675. doi: 10.1038/srep12675 (PMC4521154; doi:10.1038/srep12675)
Supplement: Supplementary Information [file srep12675-s1.doc]

Supplementary information

The Dissociation of Gefitinib Trough Concentration and Clinical Outcome in NSCLC Patients with EGFR Sensitive Mutations

Shuang Xin^a,1^, Yuanyuan Zhao^b,1^, Xueding Wang^a^, Yan Huang^b^, Jing Zhang^c^, Ying Guo^d^, Jiali Li^a^, Hongliang Li^a^, Yuxiang Ma^b^, Lingyan Chen^a^, Zhihuang Hu^b^, Min Huang^a^,*, Li Zhang^b^,*

1. **Partial validation for detection of gefitinib by LC-MS/MS**

**Chromatographic conditions:**

System: Waters Model 2695 separations system (Milford, MA, USA);

Column: Waters X-Terra RP18 3.5μm 2.1*50mm column;

Mobile phase:　acetonitrile-water (70:30, v/v) containing 0.1% formic acid;

Flow rate: 0.2mL/min;

Mean (±SD) retention time : 0.91±0.1min

Overall chromatographic run time : 3 min

**Mass-spectrometric conditions:**

Mass-spectrometric detector : Micromass Quattro Micro triple-quadrupole mass-spectrometric detector;

Software: Masslynx version V4.1 software;

Ironization mode :ES+ ;

Cone voltage: 40V ;

Collision voltage :18V ;

Gefitinib parent ion : 447.1 ; daughter ion: 128 ;

D8-gefitinib parent ion : 455.1; daughter ion :136 .

**Results:**

Supplementary Figure1a

Supplementary Figure1b

Supplementary Figure1c

Supplementary Figure1. Chromatograms of gefitinib-d8 (internal standard, top panel), gefitinib (middle panel). (a) Sample 1: blank plasma; (b) Sample 2: QC 250ng/mL gefitinib spiked in blank plasma; (c) Sample 3: patient plasma collected on days 28 prior to drug administration.

Supplementary Table1: Back-calculated concentrations from calibration curves over the concentration of 10-1000ng/mL.

| **Nominal concentration (ng/mL)** | **x±s** | **RSD (%)** | **Accuracy (%)** |
| --- | --- | --- | --- |
| **10** | 10.32±0.87 | 8.48 | 103.20 |
| **25** | 24.22±0.41 | 1.71 | 96.88 |
| **50** | 47.14±4.43 | 9.40 | 94.28 |
| **100** | 95.94±3.62 | 3.77 | 95.94 |
| **250** | 247.16±11.57 | 4.68 | 98.86 |
| **500** | 512.54±16.41 | 3.20 | 102.51 |
| **1000** | 997.28±10.94 | 1.10 | 99.73 |

Supplementary Table2: Recovery of extraction for gefitinib QC sample (n=5).

| **Nominal concentration (ng/mL)** | **Gefitnib recovery (%)** | **D8-gefitinib recovery (%)** |
| --- | --- | --- |
| **25** | 42.10±5.09 | 44.31±7.98 |
| **70** | 50.18±1.78 | 51.36±1.04 |
| **700** | 53.49±3.79 | 50.26±1.27 |

Supplementary Table3: Assessment of intra-day accuracy and precision for gefitinib QC sample (n=5).

| **Nominal concentration (ng/mL)** | **Accuracy (%)** | **Precision (%)** |
| --- | --- | --- |
| **10** | 104.00 | 8.46 |
| **25** | 104.88 | 2.55 |
| **70** | 106.48 | 3.20 |
| **700** | 107.20 | 2.27 |

Supplementary Table4: Assessment of inter-day accuracy and precision for gefitinib QC sample (n=5).

| **Nominal concentration (ng/mL)** | **Accuracy (%)** | **Precision (%)** |
| --- | --- | --- |
| **10** | 101.85 | 12.18 |
| **25** | 105.90 | 7.15 |
| **70** | 105.34 | 6.68 |
| **700** | 104.54 | 4.50 |
